# Effect of Type of Aging on Quality and Sensory Perception of Picanha (*Biceps femoris*) from Female Angus Calves

**DOI:** 10.3390/foods14132219

**Published:** 2025-06-24

**Authors:** Alberto Ortiz, María Freire, Lucía León, Francisco Javier Mesías, David Tejerina

**Affiliations:** 1 Meat Quality Area, Center of Scientific and Technological Research of Extremadura (CICYTEX-La Orden), Junta de Extremadura, Ctra. A-V, Km372, 06187 Guadajira, Spain; alberto.ortiz@juntaex.es (A.O.); lucia.leon@juntaex.es (L.L.); david.tejerina@juntaex.es (D.T.); 2 Faculty of Agriculture, University of Extremadura, Ctra. Cáceres s/n, 06071 Badajoz, Spain; fjmesias@unex.es

**Keywords:** short aging, picanha, dry-aging, wet-aging, vacuum-packaging

## Abstract

This study investigated the meat quality, sensory properties and microbiology of Angus beef after a short dry or wet aging. For that, a total of 16 *Biceps femoris* muscles from female Aberdeen Angus x Charoles calves were used. Half of these underwent a technological aging process in the carcass (dry aging) for 7 days, whilst the remaining were filleted, vacuum-packed and stored at refrigerated conditions (wet aging) for 7 days at 4 ± 2 °C. The type of aging affected the ratio of the myoglobin forms but did not translate into differences in the instrumental colour measurements. Dry aging led to minor water release after the application of a force (17.58 dry-aged vs. 31.09 wet-aged) or after cooking and yielded higher hardness and lower shear force in the Warner–Braztler shear force test compared to wet aging. Nevertheless, these differences were not appreciated at the sensory level. Higher counts of mesophilic aerobic bacteria (11.66%) and enterobacteria (3.68%) were found in samples subjected to dry aging.

## 1. Introduction

In recent times, cattle farmers have increasingly embraced the Aberdeen Angus breed. This breed is prized for its ability to enhance the quality of meat when crossbred with local and integrated breeds, primarily due to its exceptional fat infiltration capacity [1,2]. Indeed, in the United States, products stemming from this breed are renowned for their superior quality, regulated by professional associations, such as the “Certified Angus Beef” [3]. Similarly, in Spain, there exists a dedicated association advocating for the preservation and traceability of the distinctive quality products derived from the Aberdeen Angus breed—the Spanish Association of Angus cattle breeders [4]. Although, to our knowledge, there is no official census to date, it is estimated that there are more than 2000 dams in Spain, according to the Spanish Cattle Breeders Association, and in any case, the Aberdeen Angus breed is undergoing rapid growth because there is “much more demand than supply” [5]. But it is crucial to note that the excellence of beef is not solely dependent on genetic factors but also on technological aspects, such as aging. The technological process of aging is basically defined by the type of process (dry aging or wet aging) and the length of time. In the case of dry aging, beef carcasses or primal cuts are hanged and aged under aerobic conditions in chilling rooms, in which these are subjected to refrigeration temperatures, controlled relative humidity and airflow. Whereas, in wet aging, the process occurs under vacuum-packing and refrigeration conditions. The latter type of aging has been on the rise with the development of vacuum packaging [6]. Basically, the loss of product because of losses of moisture during aging and trimming (dry surface) associated with dry aging, together with the longer aging times compared to wet aging, results in lower yields of the former; this results in a more expensive process compared to wet aging, which, besides better yield, results in better processing, transport and shelf life. To the best of our knowledge and to date, studies on the comparison of the various types of aging are scarce and have focused on the evaluation of very long aging times. In this line, Kim et al. [7] tried to improve the quality of different cuts (butt, rump and sirloin) from Hanwood beef after 28 days of dry and wet aging. Ha et al. [8] also studied the effect of dry and wet aging in Australian beef loins (*Longissimus thoracis et lumborum* (LTL)) for a period of 56 days, while Di Paolo et al. [9] studied this process for up to 60 days in LTL and *Semitendinosus dorsi*. The aging times investigated in the previous studies are probably prolonged because these were carried out on pieces obtained from animals of an advanced age, which, therefore, required long aging times for tenderization. However, aging is being currently pursued for additional objectives besides tenderization, such as the enhancement of unique flavours, improving consumer-perceived eating quality [10] and, therefore, providing an added value to the aged meat [8,11]. This, therefore, may extend the aging to pieces that, due to their characteristics and the age of the animal, have not traditionally been subjected to the technological aging process or that do not require such prolonged times. Consequently, this creates the need to explore aging and the type of aging, considering short times in meat pieces such as picanha. The picanha (*Biceps femoris* muscle), also known in Spain as “*Tapilla*”, is a piece located in the lower part of the hindquarter, covered by a layer of fat and with a certain infiltration. Picanha obtained from calves is a cut with a high market acceptance, and a short technological aging process may improve its sensory characteristics and increase its added value.

On the other hand, calves are defined as animals (males and females) slaughtered at less than 12 months of age. These, for Spain, represent the largest number of bovines slaughtered [12]. Since these are young animals, the meat pieces derived from them would not need to achieve a high degree of tenderization.

In this framework, the present research study aimed to investigate the effect that the type of aging (dry vs. wet aging) to which Angus beef is subjected exerts on its main quality parameters, sensory properties and microbiology.

## 2. Materials and Methods

### 2.1. Picanha Samples

A total of 16 *Biceps femoris* muscles (meat cut known as “Picanha”) with an average weight of 2.52 kg ± 0.31 were used (Figure S1). These pieces originated from female animals of Aberdeen Angus x Charoles breeding slaughtered with a live weight between 450 and 500 kg and aged less than 12 months. Calves were reared on mother’s milk until weaning, at the age of four months. From this age until slaughter, calves were reared on conventional feed based on vegetable concentrates supplemented with vitamins and minerals. Of these, half underwent a technological aging process in the carcass (dry-aging process), with the following conditions: 2 °C ± 0.4 of average temperature in combination with a relative humidity (RH) of 80% ± 1.2 for a period of 7 days. The airflow rate of the air chamber was set at 0.35 m/s. After aging, the whole pieces were filleted for subsequent determinations. The remaining half of the pieces were filleted (2.5 cm thick steaks with an average weight of 212.10 ± 29.91 g) 24 h after slaughtering and immediately vacuum-packed and stored in refrigerated conditions (4 °C ± 2) for 7 days (wet aging). The film properties used were as follows: 52 μm of thickness, a density of 1.04 kg dm^−3^, oxygen transmission rate < 3 cc m^−2^ day^−1^ and water vapor transmission rate < 15 g m^−2^ day^−1^. At least 11 fillets were obtained from each picanha. Randomly, three of them were cooked for cooking losses and texture analysis, three were used for physical–chemical parameters, three more for microbiological analysis and two of them for sensory analysis.

### 2.2. Chemical Reagents

Buffers for the pH meter were purchased from Crison (Barcelona, Spain). Potassium phosphate buffers (0.04 M) were used to maintain a pH of 6.85 using KH_2_PO_4_ = 4.87 g and K_2_HPO_4_ = 5.94 g in 1000 mL of distilled water (Honeywell, Fluka, Madrid, Spain). 2-thiobarbituric acid, 1,1,1,3- tetraethoxypropane (TEP), 2,4-dinitrophenylhydrazine (DNPH) and HCl (12N) were acquired from Sigma Aldrich (Madrid, Spain). Water was purified by Milli-Q system (Millipore Corp., Bedford, MA, USA). All other reagents and solvents used were of a suitable grade for spectrophotometric or chromatographic analysis.

### 2.3. pH

The pH was measured directly from the “Picanha” meat cut (*Biceps femoris* muscle) after 7 days of aging (dry or wet, in each case) using a penetration electrode coupled with a temperature probe (Crison pH-meter mod. MicropH (Crison, Barcelona, Spain) 2001). Previously, the instrument was calibrated with 2-point standard buffer solutions of 7.01 and 4.01 at room temperature (20 °C ± 2).

### 2.4. Instrumental Colour

The lightness (L*), redness (a*, which evaluates the range of red to green) and yellowness (b*, yellow to blue) CIELAB space parameters were measured [13], together with the saturation index or chroma (C*), defined as C = (a*^2^ + b*^2^)*0.5, and Hue angle (Hº), defined as arctg (b*/a*) [14], by means of a Minolta CR-400 colourimeter (Minolta Camera, Osaka, Japan), with illuminant D65, a 0° standard observer and a 2.5 cm port/viewing area. The blooming time was 15 min. In order to maximize product variability, measurements were taken at five randomly selected points in each sample (the steak) and then averaged.

### 2.5. Myoglobin and Chemical Forms

Myoglobin content and its chemical forms were determined according to the method given by Pujol et al. [15] and Tang et al. [16]. Samples were mixed with 40 mM phosphate buffer (KH_2_PO_4_ = 4.87 g and K_2_HPO_4_ = 5.94 g in 1000 mL of distilled water) (pH 6.85) by homogenizer (IKA ULTRA-TURRAX Homogenizer T-25) at 12,000× *g* for 1 min. Then, centrifugation (2500× *g*/10 min/4 °C) and filtration with Whatman Nº1 filter were performed to remove protein and fat content. After that, the absorbance at 503/525/557/582/700 nm was measured by ultraviolet–visible spectrophotometer (Agilent Cary 60). Myoglobin content and myoglobin composition were determined by the following equation:

Myoglobin content:

$$Mb\left[\frac{mg}{g\;product}\right]=\left(\lambda 525-\lambda 700\right)\times \frac{1\;mMMb}{7.6}\times \frac{\frac{1\;mmol}{L}}{mM}\times \frac{17\;gMb}{1\;mmolMb}\times \frac{0.03\;(L)}{sample\;weight(g)}\times \frac{1000\;mg}{1\;g}$$

where A is the absorbance at λ nm, 7.6 is the millimolar extinction coefficient at 525 nm, and 17 (kDa) is the average molecular weight value of Mb.

Whilst the chemical forms were determined as follows:

$$\mathrm{MB}\;\left[DMb\%\right]=\left(-0.543\;\left(\frac{\lambda 582}{\lambda 525}\right)+1.594\;\left(\frac{\lambda 557}{\lambda 525}\right)+0.552\;\left(\frac{\lambda 503}{\lambda 525}\right)-1.329\right)\times 100$$

$$\mathrm{OxyMb}\left[OMb\%\right]=\left(0.722\;\left(\frac{\lambda 582}{\lambda 525}\right)-1.594\;\left(\frac{\lambda 557}{\lambda 525}\right)-0.552\;\left(\frac{\lambda 503}{\lambda 525}\right)+2.599\right)\times 100$$

$$\mathrm{MetMb}\left[MMb\%\right]=\left(-0.159\;\left(\frac{\lambda 582}{\lambda 525}\right)-0.085\;\left(\frac{\lambda 557}{\lambda 525}\right)+1.262\left(\frac{\lambda 503}{\lambda 525}\right)-0.520\right)\times 100$$

### 2.6. Dry Matter and Water Losses

Dry matter (DM) was determined according to the AOAC method [17] and expressed as g/100 g. The water holding capacity (WHC) was assessed by measuring the amount of free water released (g/100 g) after centrifugation at 1800× *g* for 3 min [18]. The cooking losses were calculated as the difference before and after cooking a vacuum-packed 2.5 cm thick steak by immersing it in a water bath at 80 °C for 45 min (g/100 g).

### 2.7. Oxidative Status

Lipid oxidation, determined as thiobarbituric acid reactive substances (TBA-RS), was evaluated by the 2-thiobarbituric acid (TBA) method [19]. TBA-RS values were calculated from the standard (1,1,1,3 - tetraethoxypropane, TEP) curve and expressed as µg malondialdehyde (MDA)/g.

Protein oxidation was determined by measuring the carbonyl groups generated upon incubation with 2,4-dinitrophenylhydrazine (DNPH) in 2N HCl, as described in the method of Oliver et al. [20].

### 2.8. Fatty Acid Profile

The fatty acid profile was calculated from the intramuscular fat extracted previously, following the method described by Folch et al. [21]. The extraction was as determined by Contador et al. [22]. One microlitre of the sample was injected into a gas chromatograph (model 4890 Series II; Hewlett-Packard, Palo Alto, CA, USA) equipped with a split/split-less injector and a flame ionization detector. FAMEs were separated on a Carbowax™ fused silica capillary column (30 m × 0.25 mm id; 0.25 μm film thickness; Ohio Valley, Marietta, OH, USA). The oven temperature was held at 200 °C. The injector and detector were set at 250 °C. The carrier gas was nitrogen at 1.8 mL min^−1^. The identification of individual FAME was based on a standard mixture of 37 Component FAME Mix (Sigma–Aldrich, Supelco 37 Component FAME Mix- CRM47885, St. Louis, MO, USA). The amount of each fatty acid and of the different fatty acid groups was calculated on the total of fatty acids detected and expressed as g/100 g of fatty acid methyl esters (FAMEs).

### 2.9. Texture Analysis

Both texture profile analysis (TPA) and Warner–Bratzler shear force test (WBSFT) were carried out on cooked meat, as previously described for cooking losses determination.

TPA was performed with a 20% deformation to assess the contribution of myofibrillar structures without connective tissue intervention to textural properties [23] by means of a TA XT-2i Texture Analyser (Stable Micro Systems Ltd., Surrey, UK). Thus, 1 cm^3^ samples were compressed to the above-mentioned percentage of their original height using a 20 mm diameter (P/20) flat plunger (crosshead speed of 2 mm/s through a 2-cycle sequence) in parallel direction to the muscle fibres. From the resulting force–deformation curves, the following parameters were measured: hardness (N), springiness (cm), cohesiveness (adimensional), gumminess (N cm s^2^), chewiness (N cm s^2^), resilience (adimensional) [24].

For the purpose of performing a Warner–Bratzler test, samples were prepared as 15 × 30 × 5 mm slices (width × length × thickness). Samples were cut with a Warner–Bratzler blade (HDP/BS) in perpendicular direction to the muscle fibres. The maximum shear force (N/cm^2^) was measured to cut samples. For both TPA and shear force tests, a 25-kN load cell was used. Instrumental determinations were repeated 8 times per sample, and results were averaged.

### 2.10. Microbiological Analysis

To conduct the microbiological determinations, 10 g of the sample was collected using sterile procedures and then mixed with 90 mL of peptone water using a Stomacher 400^®^ Circulator (Seward, Alaska). Following that, successive dilutions were prepared in sterile peptone water. Afterwards, 1 mL of the suitable dilutions was spread onto the selective agar plate.

Mesophilic aerobic bacteria counts were determined by means of standard Agar Count Plates (Merk 1.07881) (30 °C for 72 h of incubation). *Clostridium perfringens* counts were determined by incubation on a TSC Agar (Merk 1.20426) at 37 °C for 24 h.

*Cl. Sulfitoreductors* counts were determined by means of SPS Agar Count Plates after incubation for 24–48 h at 37 °C. Enterobacteria counts were determined by incubation at 37 °C during 24–48 h on Chromocult^®^ Agar (Merck, Darmstadt, Germany) (Merck, 1.10426). Halotolerant bacteria were determined by incubation at 30 °C for 72 h on Salted Mannitol Agar.

The presence of *Salmonella* spp. and *Listeria monocytogenes* was determined in 25 g of sample.

Microbiological results were expressed as log_10_ colony-forming units (CFU)/g, except for *Salmonella* spp. and *Listeria monocytogenes,* which were expressed as presence or absence.

### 2.11. Sensory Evaluation

A sensory analysis was conducted by 10 trained judges using a quantitative–descriptive analysis method; the judges previously signed the informed consent for the use of humans in sensory analysis. Appropriate protocols for protecting the rights and privacy of all participants were utilized during the execution of the research. All panellists had been previously screened for sensory acuity and were experienced in the sensory analysis of meat products. All training (4 training sessions) and formal (6 sessions) evaluation sessions were conducted by the same panellists and carried out at room temperature in a sensory room equipped with white fluorescent lighting. Water (100 mL) at room temperature was provided to the panellists between samples.

**Figure 1:**
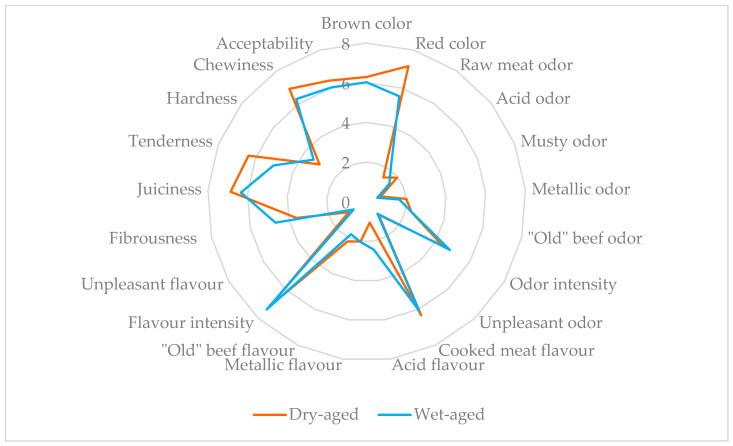
Effect of type of aging on sensory assessment of Angus beef.

Firstly, a list of descriptors for both products was provided to each panellist, and then, the redundant descriptive terms were removed using dichotomous responses; the selected attributes were evaluated using an unstructured scale of 0–10, with verbal anchors ‘little’ and ‘very much’. The panel was trained using three random cubes of meat from each sample, selected to clarify aroma, flavour, and texture attributes relevant to aged meat. From these reference samples, two visual descriptors, seven odour descriptors, six flavour descriptors, five texture attributes and global acceptability were defined (Figure 1).

### 2.12. Statistical Analysis

One-way ANOVA test was used to study the effect of the type of aging (dry or wet aging) on pH, colour, dry matter and water losses, oxidative status, fatty acid profile, instrumental texture, and microbiological counts, according to the following model:

Y_ij_ = µ + A_i_ + e_i(j)_

where Y_ij_ refers to the variable under consideration, µ is the mean value, A_i_ is the type of aging and e_i(j)_ is the residual error. Statistical significance was set at *p* ≤ 0.05. All experiments were performed at least three times and averaged. Values were expressed as mean ± standard error.

## 3. Results and Discussion

### 3.1. Effects of the Type of Aging on pH, Colour, Water Losses and Oxidative Status

The type of aging exerted a significant effect on pH (Table 1). Dry-aged samples yielded a higher pH value than the wet-aged ones, which is consistent with previous scientific literature [25,26]. In any case, the pH value was between 5.4 and 5.6, a typical pH value for aged beef [25]. On the other hand, differences in the chemical forms of myoglobin were observed (Table 1). Specifically, wet-aged samples showed higher values of oxymyoglobin and lower values of metamyoglobin and deoxymyoglobin compared to dry-aged ones. The stability of the chemical colour of the meat is the result of the reduction and autoxidation of myoglobin. Thus, the higher microbial load and the presence of oxygen in dry-aged samples could be responsible for the higher formation of metamyoglobin in these samples [27] and their minor oxymyoglobin values with respect to the wet-aged ones [28]. Even though the scientific literature points to brown discolouration because of metamyoglobin formation, this remained below 40%, the critical limit above which meats become unsalable, according to Renerre and Labas [29], which may explain why the differences between the chemical forms of myoglobin did not translate into differences at the level of instrumental colour (Table 1). In addition, although the scientific literature has reported differences in instrumental colour associated with the type of aging, these differences depend on the aging time [30]. So, the aging time considered in this study may probably not be long enough to have an impact on instrumental colour. These results are in line with Di Paolo et al. [9], who did not report significant differences in lightness, redness or yellowness due to aging type in both LTL and *Semitendinosus* Charolais beef cuts throughout the entire range of aging time studied (from 2 to 60 days).

Regarding water losses (Table 1), a higher amount of water release was observed after the application of a force—centrifugal force—as well as after cooking in the samples subjected to wet aging. During dry aging, proteolysis and collagen modifications may alter the muscle structure, significantly influencing ion–protein interactions and reducing the diameter of microcapillaries, which decreases the ability of proteins to retain water [31]. This causes large water loss during dry aging, leading to a lower volume available to be released when an external force is exerted or after cooking in dry-aged samples.

In terms of oxidative status, no differences were observed for either malondialdehyde content or nanomol carbonyls/mg protein content with respect to the type of aging (Table 1). Overall, the lipid oxidation values were like those found by Di Paolo et al. [9] in LTL and *Semitendinosus* Charolais beef cuts subjected to dry aging for similar times. In any case, the malondialdehyde values were far from exceeding the value of 2 µg/g, above which the eating quality could be compromised due to off-flavour and taste [32]. Although dry aging may promote higher intramuscular lipid oxidation because of the exposure to oxygen [33], the lack of difference with respect to wet aging could be due to several factors. First, the short aging time proposed in the present study. In fact, Di Paolo et al. [9] did not observe a higher malondialdehyde content in dry-aged cuts compared to wet-aged ones until after 15 days. Secondly, the fat coverage of the piece may have exerted a protective effect against oxygen exposure and lipid oxidation in the case of dry aging. In the case of wet-aged cuts, the low malondialdehyde values could be due to the protection offered by the vacuum packaging, which reduces the transmission of oxygen to the samples, preventing the oxidation of the fat [33]. In this line, Ba et al. [34] reported values below 0.40 µg of malondialdehyde/g even after 28 days of vacuum chiller aging in LTL and *Semitendinosus* muscles from Korean native cattle beef. With respect to the protein carbonyl content, the values obtained could be considered low and similar to those obtained by Zhang et al. [26] in dry-aged lamb. As for lipid oxidation, the short aging time together with the fat coverage of the piece and packaging for dry- and wet-aging processes, respectively, may have protected the samples against a high-oxygen atmosphere or the reactive agents, such as light, preventing the increase of carbonyls [35,36].

### 3.2. Effect of the Type of Aging on Fatty Acid Profile

The fatty acid values during different types of aging are presented in Table 2. The most prevalent fatty acid identified was oleic acid (C18:1 n-9), followed by stearic acid (C18:0). Turning to the main fatty acid groups, both monounsaturated fatty acid (MUFA) and saturated fatty acid (SFA) groups summed up to similar proportions of around 45%, with PUFA being in the minority.

With respect to the impact exerted by the type of aging on the lipid profile, only a significantly lower proportion of the PUFA group was observed in the wet-aged samples with respect to the dry-aged ones. This is primarily explained by the same trend followed by linoleic acid (C18:2 n-6), the main fatty acid of this group, and may be related to lipolysis and lipid oxidation processes. Even though lipid oxidation did not show significant differences according to the type of aging, the lower proportion of PUFA in dry-aged samples may indicate an initial state of oxidation of these lipids [37] because of their exposure to oxygen during this type of aging. Indeed, Di Paolo et al. [9] recently reported a lower PUFA proportion in dry-aged LTL and *Semitendinosus* Charolais beef cuts with respect to the wet-aged ones after 30 days of aging, which they associated with the oxidative state. Conversely, the barrier to oxygen transmission exerted by the packaging in the case of wet-aged samples may have contributed to preserving the oxidative state of the latter [33] and, consequently, to lower PUFA degradation.

Other individual fatty acids showed some significant differences, but these did not reflect a clear trend associated with the type of aging and may be caused by the inherent variability in the samples.

### 3.3. Effects of the Type of Aging on Textural Properties

Table 3 compiles the results from the TPA and Warner–Braztler shear force tests according to the type of aging. In general, the texture parameters from TPA yielded higher values in dry-aged samples with respect to wet-aged ones, especially for hardness (5.62 vs. 1.50), gumminess (2.59 vs. 0.91) and chewiness (2.04 vs. 0.58). TPA with low deformation percentages, as the one considered in this study, is associated with the behaviour of myofibrillar fibres without the intervention of connective tissue and with WHC parameter [38,39]. In our study, dry-aged samples showed higher pH values, as well as lower WHC and cooking loss compared to wet-aged ones, which may be related to greater muscle integrity [40], which may indicate less degradation of myofibrillar muscle fibres. This behaviour was opposite to the trend reported in other studies [9]. This may be due to the type of meat muscle analysed, since the effect of the type of aging on the final characteristics of the meat depends on the anatomical cut [7], fat content and marbling [6]. On the other hand, higher shear force was obtained in wet-aged samples compared to dry-aged ones in the Warner–Braztler shear force test (Table 3), which is consistent with previous studies carried out on sirloin meat cuts [41].

### 3.4. Effect of the Type of Aging on the Microbiological Profile

The type of aging influenced the population of mesophilic aerobic bacteria and enterobacteria (Table 4) (*p* ≤ 0.05).

Specifically, higher counts of both were found in samples subjected to dry aging. The exposure of meat to the environment may have enhanced microbial contamination and increased the surface microbial development compared to wet aging, which provides better control of microbial development due to the protection offered by the vacuum packaging [42]. In the case of dry-aged samples, the values exceeded the limit established by the European Food Safety Authority (EFSA) (<6.7 log CFU/g). However, as the surface of this product is removed prior to consumption, it may be considered safe if properly cooked, as indicated by EFSA. On the other hand, in general terms, the populations of these species were higher than those reported by Di Paolo et al. [9] in dry- or wet-aged Charolais beef, considering longer aging times than those studied in the present study. These discrepancies could be due to hygienic conditions during the carcass’ handling or slicing and the technological process of aging, since the population of mesophilic aerobic bacteria as well as enterobacteria have been reported as indicators to evaluate the hygienic conditions of the meat [43].

*Clostridium perfringens* and *sulfitoreductors* remained below the detection limits of 1 Log UFC per gram of sample (Table 4). Finally, pathogenic bacteria such as *Salmonella* and *Lysteria monocytogenes* were not detected in either group of samples, dry- or wet-aged ones (Table 4), which is in line with Di Paolo et al. [9]. The appearance of these pathogens has been associated with the conditions prevalent during the slaughter and processing of the carcasses [44], so their non-detection denotes the correct tasks in the steps prior to the technological processing of the samples.

### 3.5. Effect of the Type of Aging on Sensory Assessment

The results of the sensory panel evaluation of the cooked Angus picanha according to the type of aging are shown in Figure 1. It is noticeable that the type of aging did not affect sensory attributes, so the acceptability of both types of aging was the same. Regarding the colour parameters (brown and red), it was expected that the panellists would give similar scores for the colour parameters due to the lack of differences observed at the instrumental level. Similarly, the lack of differences in oxidative status associated with aging type could have explained the lack of differences in sensory attributes related to odour attributes (raw meat, acid, musty, metallic, “Old” beef, intensity and unpleasant odour). In contrast, flavour attributes (cooked meat, acid, metallic, “Old” beef flavour intensity, unpleasant flavour) were similar, showing different values in metallic and “Old beef” flavour notes, which could be influenced by the aging process itself. On the other hand, the lack of differences in texture-related parameters, especially hardness and tenderness, contrasts with the results obtained from instrumental texture analysis, since sensory evaluation is generally correlated with parameters derived from instrumental texture analysis [45]. This could be explained in part because intramuscular fat content is the main factor responsible for variations at the sensory level [34], for which there were obviously no differences (2.52 ± 0.26 dry-aged vs. 2.54 ± 1.04 wet-aged). As for the scientific literature related to the sensory impact of the type of aging (dry aging vs. wet aging), it has reported inconsistent results. Campbell et al. [46] concluded a greater tenderness and juiciness, as reported by the panellists in LTL subjected to dry aging versus wet aging after 14 days. Similarly, Ha et al. [8] reported higher consumer scores for dry-aged Australian beef loins with respect to those subjected to wet aging for eating attributes, like tenderness, juiciness, flavour and overall liking. Conversely, Smith et al. [47] reported that the aging treatment and aging period (comprised between 14 and 35 days) did not affect consumer sensory attributes. It is likely that the type of meat cut, aging time and/or conditions may affect its impact at the sensory level; hence, it is of interest to evaluate the impact of technological processing and conditions for each meat cut.

## 4. Conclusions

The results of this research shed some light on how technological processing can impact the quality, microbiology and sensory properties of the picanha meat cut from the Aberdeen Angus breed. The results obtained indicated that the type of aging may have an impact on some quality traits, especially those related to the ratio of different myoglobin forms, the water losses and instrumental texture. Nonetheless, the results showed that the variations at the physical–chemical level were not detected by the sensory panel, so they will probably not be perceived by the final consumer either. Therefore, in view of these results, for short times, wet aging would meet the quality expectations of consumers for aging meat as long as the aging is performed under more controlled conditions, which could have advantages in terms of shelf life and greater ease in terms of product distribution and logistics compared to dry aging.

## Figures and Tables

**Table 1:** Effects of the type of aging on colour, water losses and oxidative status of Angus beef.

	Dry-Aged	Wet-Aged	*p* Value
pH	5.60 ± 0.02	5.44 ± 0.07	***0.000***
Instrumental colour			
CIE-L*	40.20 ± 0.60	41.80 ± 0.72	0.125
CIE-a*	25.40 ± 0.24	24.50 ± 0.72	0.293
CIE-b*	13.20 ± 0.28	12.60 ± 0.55	0.376
Chroma	28.60 ± 0.33	28.30 ± 0.80	0.761
Hue	27.50 ± 0.32	28.00 ± 0.64	0.469
Chemical colour			
Mb (mg/g)	7.20 ± 0.24	7.88 ± 0.16	***0.039***
Deoxy (%)	12.83 ± 0.35	7.04 ± 0.57	***0.000***
Oxy (%)	50.19 ± 1.60	63.85 ± 1.87	***0.000***
MetMb (%)	35.27 ± 0.83	23.97 ± 0.92	***0.000***
Dry matter and Water losses			
DM (g/100 g)	26.01 ± 0.22	26.15 ± 0.73	0.857
WHC (g of water released/100 g)	17.58 ± 0.42	31.09 ± 0.42	***0.000***
Cooking loss (g of water released/100 g)	33.49 ± 0.84	38.21 ± 0.66	***0.001***
Oxidative status			
µg MDA/g	0.42 ± 0.03	0.36 ± 0.03	0.145
Nanomol carbonyls/mg protein	2.38 ± 0.09	2.36 ± 0.06	0.875

Values are expressed as means ± standard error. CIE-L*: lightness, CIE-a*: redness, CIE-b*: yellowness measured in the CIE Lab space, Mb: myoglobin, Deoxy: deoxymyglobin, Oxy: oxymyglobin, MetMb: metamyoglobin, DM: dry matter, WHC: water holding capacity, MDA: malhondialdehyde.

**Table 2:** Effects of the type of aging on the fatty acid profile from intramuscular fat of Angus beef.

	Dry-Aged	Wet-Aged	*p* Value
g/100 g FAMEs			
C12:0	0.06 ± 0.01	0.07 ± 0.01	0.383
C14:0	2.36 ± 0.20	2.88 ± 0.18	0.079
C16:0	26.02 ± 0.71	27.20 ± 0.45	0.194
C16:1	4.04 ± 0.19	5.07 ± 0.39	***0.038***
C17:0	1.00 ± 0.02	0.84 ± 0.03	***0.003***
C17:1	0.61 ± 0.02	0.68 ± 0.03	0.113
C18:0	15.28 ± 0.28	13.20 ± 0.42	***0.002***
C18:1 n-9	42.43 ± 0.65	42.68 ± 1.36	0.872
C18:2 n-6	4.88 ± 0.21	5.80 ± 0.33	***0.043***
C18:3 n-3	0.14 ± 0.01	0.08 ± 0.01	***0.001***
C20:0	1.16 ± 0.12	0.40 ± 0.05	***0.000***
C20:1 n-9	0.73 ± 0.04	0.46 ± 0.04	***0.001***
PUFA	5.02 ± 0.21	5.89 ± 0.33	0.052
MUFA	47.82 ± 0.60	48.90 ± 1.54	0.529
SFA	45.87 ± 0.51	44.59 ± 0.66	0.153

Values are expressed as means ± standard error. FAMEs: fatty acid methyl esters, C12:0: lauric acid, C14:0: myristic acid, C16:0: palmitic acid, C16:1: palmitoleic acid, C17:0: margaric acid, C17:1: margaroleic acid, C18:0: stearic acid, C18:1 n-9: oleic acid, C18:2 n-6: linoleic acid, C18:3 n-3: linolenic acid, C20:0: arachidic acid, C20:1: gadoleic acid, PUFA: polyunsaturated fatty acids, MUFA: monounsaturated fatty acids, and SFA: sum of the polyunsaturated, monounsaturatted and saturated fatty acids.

**Table 3:** Effects of the type of aging on textural properties of Angus beef.

	Dry-Aged	Wet-Aged	*p* Value
Compression Test (20% compression)			
Hardness (N^2^)	5.62 ± 0.35	1.50 ± 0.22	***0.000***
Springiness (cm)	0.76 ± 0.02	0.68 ± 0.02	***0.018***
Cohesiveness	0.63 ± 0.03	0.57 ± 0.01	0.131
Gumminess (N cm s^2^)	2.59 ± 0.13	0.91 ± 0.14	***0.000***
Chewiness (N cm s^2^)	2.04 ± 0.09	0.58 ± 0.07	***0.000***
Resilience	0.46 ± 0.03	0.42 ± 0.01	0.191
Warner–Braztler shear force test			
Shear force (N)	44.76 ± 0.29	52.5 ± 1.94	0.003

Values are expressed as means ± standard error.

**Table 4:** Effects of the type of aging on microbiology (log10 CFU/g) of Angus beef.

	Dry-Aged	Wet-Aged	*p* Value
Mesophilic aerobic bacteria	6.84 ± 0.11	6.04 ± 0.05	***0.000***
*Cl. perfringens*	<1	<1	-
*Cl. sulfitoreductors*	<1	<1	-
Enterobacteria	5.43 ± 0.05	5.23 ± 0.07	0.059
Halotolerant bacteria	2.29 ± 0.10	1.80 ± 0.28	0.133
*L. Monocytogenes*	Absence	Absence	-
*Salmonella*	Absence	Absence	-

Values are expressed as means (log10 CFU/g) ± standard error. The limit of detection for the method for microbiological analysis was 1 Log CFU.

## Data Availability

The original contributions presented in this study are included in the article. Further inquiries can be directed to the corresponding author.

## Supplementary Materials

The following supporting information can be downloaded at: https://www.mdpi.com/article/10.3390/foods14132219/s1, Figure S1: Picanha, *Biceps femoris*.
