# Supplementary figures and images for: Effect of Type of Aging on Quality and Sensory Perception of Picanha (Biceps femoris) from Female Angus Calves
Source: Foods. 2025 Jun 24;14(13):2219. doi: 10.3390/foods14132219 (PMC12249328; doi:10.3390/foods14132219)

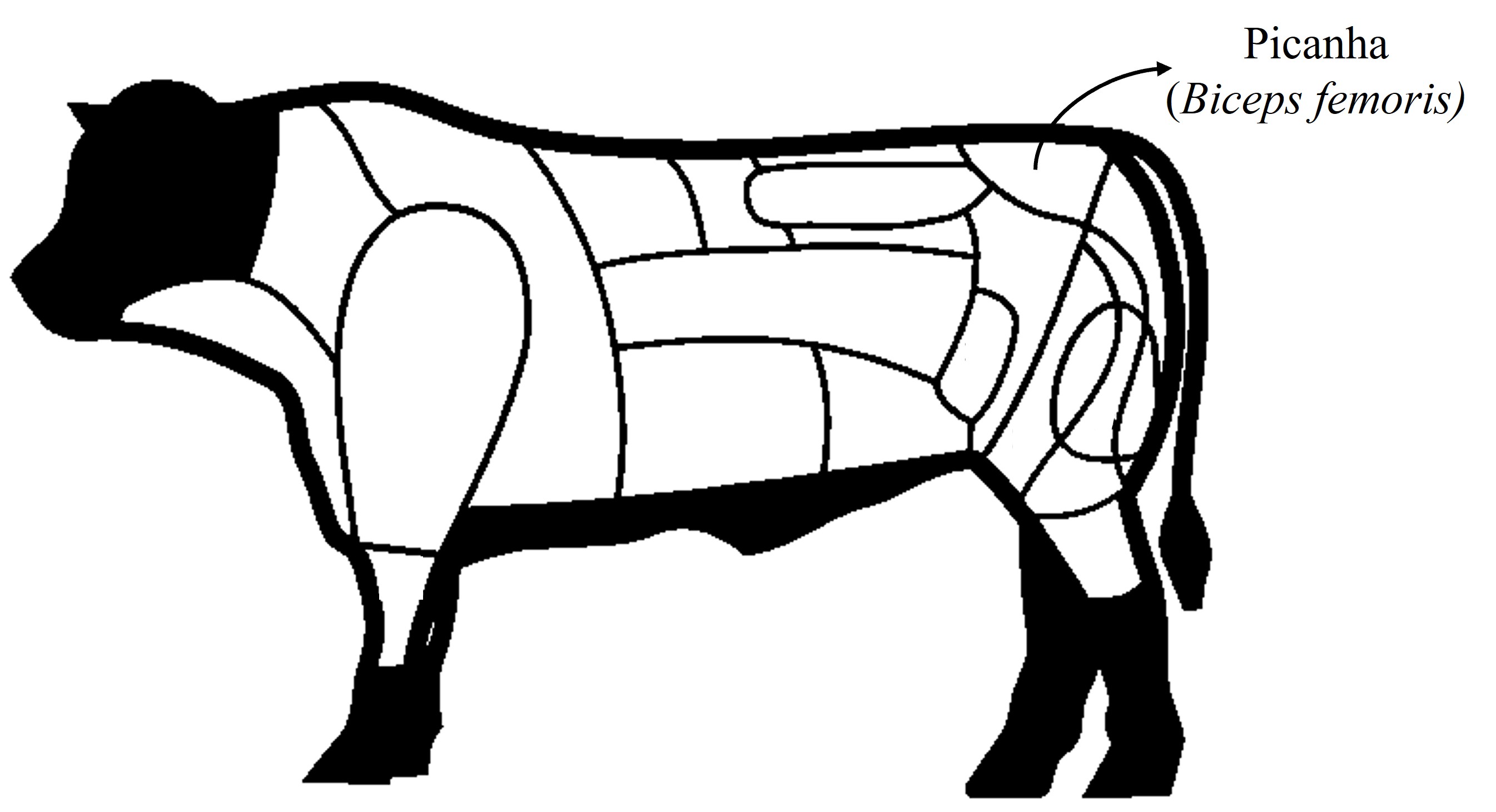

Supplement: Supplementary file 1 [file foods-14-02219-s001.zip › foods-3671308-supplementary.jpg]
